# Genetic Contribution of Synthetic Hexaploid Wheat to CIMMYT’s Spring Bread Wheat Breeding Germplasm

**DOI:** 10.1038/s41598-019-47936-5

**Published:** 2019-08-26

**Authors:** Umesh Rosyara, Masahiro Kishii, Thomas Payne, Carolina Paola Sansaloni, Ravi Prakash Singh, Hans-Joachim Braun, Susanne Dreisigacker

**Affiliations:** 1 Global Wheat Program, International Maize and Wheat Improvement Center (CIMMYT), Carretera México-Veracruz Km 45, El Batán, Texcoco, C.P. 56237 Mexico; 2 Wheat Germplasm Bank & International Wheat Improvement Network, International Maize and Wheat Improvement Center (CIMMYT), Carretera México-Veracruz Km. 45, El Batán, Texcoco, C.P. 56237 Mexico; 3 Genetic Resource Program, International Maize and Wheat Improvement Center (CIMMYT), Carretera México-Veracruz Km. 45, El Batán, Texcoco, C.P. 56237 Mexico

**Keywords:** Agricultural genetics, Plant genetics

## Abstract

Synthetic hexaploid (SH) wheat (AABBD’D’) is developed by artificially generating a fertile hybrid between tetraploid durum wheat (*Triticum turgidum*, AABB) and diploid wild goat grass (*Aegilops tauschii*, D’D’). Over three decades, the International Maize and Wheat Improvement Center (CIMMYT) has developed and utilized SH wheat to bridge gene transfer from *Ae*. *tauschii* and durum wheat to hexaploid bread wheat. This is a unique example of success utilizing wild relatives in mainstream breeding at large scale worldwide. Our study aimed to determine the genetic contribution of SH wheat to CIMMYT’s global spring bread wheat breeding program. We estimated the theoretical and empirical contribution of D’ to synthetic derivative lines using the ancestral pedigree and marker information using over 1,600 advanced lines and their parents. The average marker-estimated D’ contribution was 17.5% with difference in genome segments suggesting application of differential selection pressure. The pedigree-based contribution was correlated with marker-based estimates without providing chromosome segment specific variation. Results from international yield trials showed that 20% of the lines were synthetic derived with an average D’ contribution of 15.6%. Our results underline the importance of SH wheat in maintaining and enhancing genetic diversity and genetic gain over years and is important for development of a more targeted introgression strategy. The study provides retrospective view into development and utilization of SH in the CIMMYT Global Wheat Program.

## Introduction

Bread wheat (*Triticum aestivum L*.; hexaploid genome = AABBDD) naturally evolved via natural hybridization between wild goat grass *Aegilops tauschii* (DD) and a cultivated emmer plant *T*. *turgidum* L. ssp. *dicoccon* (Schrank) Thell. (2n = 28; AABB, a progenitor of modern durum wheat) around 8,000 years ago^1,2^. Thus, it consists of three diploid progenitor genomes, AA from *Triticum urartu*, BB from an unknown species (suggested to be section *Sitopsis* to which *Aegilops speltoides* belongs), and DD from *Ae*. *tauschii*^3,4^. The current form of the *Ae*. *tauschii* genome (denoted here as D’) is expected to be similar to the original progenitor genome (denoted as D) that led to development of bread wheat. Studies have also suggested that the D genome originated from a homoploid ancestor (derived from the hybridization between A and B diploid progenitors) about five million years ago^5,6^. The current bread wheat D genome has limited genetic diversity due to (a) hexaploid wheat is expected to have evolved from a few hybridization events with *Ae*. *tauschii* (b) there has been limited gene flow from *Ae*. *tauschii* to bread wheat, both naturally being highly self-pollinated species with inbreeding coefficients ≥90% and (c) intense human selection of bread wheat led to further depletion of diversity^7^.

With the development of next-generation sequencing technologies, high-density genome profiling of plant material has become feasible and relatively cost-effective^8,9^. These new genotyping technologies have already been effectively used to characterize the genetic diversity in bread wheat^10–12^. The limited genetic diversity of the D genome in bread wheat is always reflected by less SNP polymorphisms observed when compared to A and B genomes^10,13–15^. The D’ genome is assumed to be structurally similar to the D genome, however as it has not gone through the same extent of natural and human selection, which results in higher SNP diversity^16^.

Although the introgression of D’ genome via SH wheat to synthetic derivative lines can introduce novel variation for traits of interest^17–19^, it may contain unfavorable or detrimental genetic load with complex genetics of pleiotropy and epistatis^20^. The undesirable component of D’ can be reduced by recurrent back or top crossing with elite germplasm and selection. However, in the process of retaining the desirable D’ alleles, undesirable alleles at closely linked loci may still be present in synthetic derived lines, causing what is also called ‘linkage drag’ or ‘genetic load’^21^.

Similar to the D genome, nucleotide diversity in the A and B genomes of bread wheat is also substantially reduced compared to their ancestral progenitors^22^. The A and B genomes from bread wheat do not reflect or represent the genetic diversity existing in durum wheat^23,24^. However, bread and durum wheat being both cultivated and selected over generations, the contribution from the durum A and B genomes when introgressed into synthetic derivative lines are not considered detrimental. Thus, SH wheat (AABBD’D’) can boost the genetic diversity in all three genomes, as has been well documented^25–27^.

*Ae*. *tauschii* harbors substantial variation for many biotic and abiotic stress tolerance traits that are relevant in wheat breeding^28^. The first attempts to reproduce the bread wheat original crosses were made in the 1940s in Japan^29^ and US^30,31^. These attempts led to the development of the first SH wheat^28^. To upscale impact globally, CIMMYT started to explore the value of wide crosses and the development of SH wheat to increase D genome diversity in the 1980s. The artificial re-creation of bread wheat from improved tetraploid durum wheat and accessions of *Ae*. *tauschii* was tested and deployed at larger scale^32^. The use of improved tetraploid wheat was important to success as two out of the three genomes have already been selected for desirable traits. A cross with wild tetraploid wheat species usually leads to tall synthetics with very undesirable agronomic properties.

When desirable alleles for a trait of interest are considered limited in the current elite germplasm pool, SH wheat is one of first materials to be additionally evaluated in the CIMMYT Global Wheat Program and other bread wheat breeding programs^28,33^. Synthetic hexaploid wheat have provided valuable diversity for traits related to agronomic and physiological features^33–39^, abiotic stress tolerance^33,36,40–43^, biotic stress resistance^33,35,41,44–60^, and grain quality^61–63^. To date, the CIMMYT wide-crosses program has developed more than 1,524 SH wheat since the 1980s and has generated thousands of crosses with bread wheat^33^. Synthetic derivative lines have been selected as parents for mainstream breeding, with rigorous selection resulting in advanced lines with excellent performance for yield and other traits. Synthetic derivative lines have been selected as candidates in international yield trials, which are disseminated every year globally. Over 80 synthetic derivative lines have also been released as cultivars and are widely grown^64,65^. Linkage drag may discourage wheat breeders to use SH wheat in their breeding programs^21^. Pre-breeding efforts are required to retain the desirable variability of the D’, A and B genomes and reduce the undesirable genetic load. Studies on the genetic contribution of the durum A and B genomes and *Ae*. *tauschii* D’ genome diversity that remain in synthetic derivatives used and retained in a breeding program are limited. The objectives of our study were to: (a) estimate the genetic contribution of the D’ genome via DartSeq® markers in a set of selected synthetic derivative lines and determine its correlation with the expected theoretical contribution calculated using ancestral pedigree information, (b) test if the genetic contribution is disproportionate in different parts of the genome, and (c) measure the theoretical contribution of SH wheat within the best CIMMYT advanced breeding lines distributed in international yield trials over the last 19 years.

## Results

### Marker-estimated contribution vs. the theoretical contribution of the D’ genome in the synthetic derivative lines

After applying the various data filters (see methods), a total of 2,669 D genome specific markers (975 PAV and 1,694 SNPs) were recovered for further analysis (Supplementary Figs S1, S2). The frequency of a D genome specific marker, with a differential frequency between the synthetic hexaploid wheat *(shw)* and *bread wheat (bw)* populations, was significantly higher than the A and B genome specific markers, with differential frequencies between the *durum wheat (dw)* and *bw* populations (Supplementary Fig. S2). The population allele frequency difference was set to 0.3 falls in 80 percentiles of the distribution. Therefore, only the D genome specific markers were used to estimate of D’ contribution from *Ae*. *tauschii* to the synthetic derivative lines. The number of differential DarSeq® markers on the A and B genome was considered to be too low to precisely estimate the contribution of the A and B genomes of durum wheat to the synthetic derivative lines.

**Figure 1:**
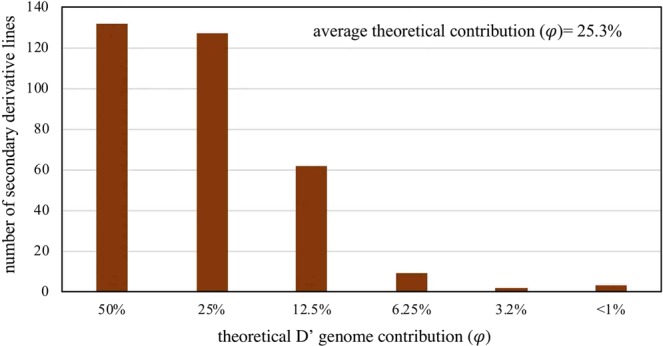
Theoretical D’ genome contribution in a set of 253 selected synthetic derivative lines (SD).

**Figure 2:**
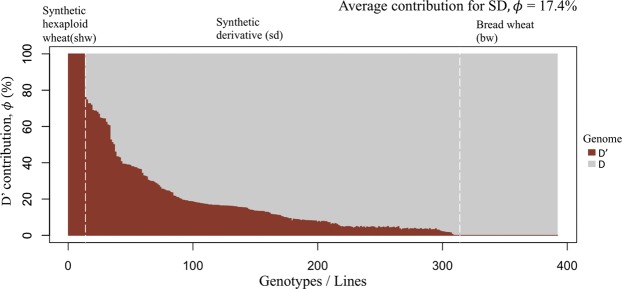
Marker-based estimate of the contribution of D’ genome of *Ae. tauschii* in a set of 253 selected synthetic derivative lines (SD).

The average theoretical contribution of the D’ genome to the 253 synthetic derivatives in our study was 25.3% (Fig. 1). In contrast the marker-estimated contribution of the D’ genome was 17.4% (Fig. 2). Thus, the marker-based estimate was 7.9% lower than the theoretical estimate. However, there was a high correlation between both estimates (r = 0.88**) (Supplementary Fig. S3). The majority of the synthetic derivative lines showed a marker-estimated contribution,, less than expected. The synthetic derivative lines of generation 1 and 2 had on an average a lower D’ contribution (*ϕ*) compared to expectation (*φ* = 12.5 to 25.0%). On the other hand, synthetic derivative lines of subsequent introgression cycle maintained a somewhat higher D’ contribution than expected (Figs 1, 2, Supplementary Fig. S3). The results indicate a relatively rapid selection against the undesirable genome segments of *Ae*. *tauschii* during the first introgression cycles but a residual D’ contribution due to linkage drag in later introgression cycles.

**Figure 3:**
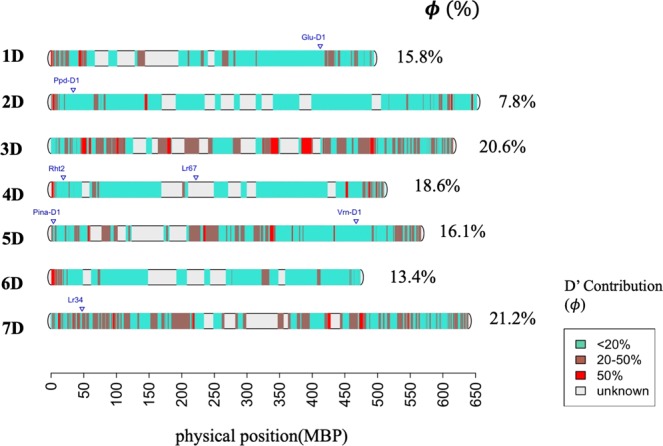
Most likely genomic regions with a D’ genome contribution. Physical positions are based in Chinese spring wheat genome sequence RefSeq v1.0.

In a second step, we looked how the contribution of D’ genome to the synthetic derivative lines is distributed across the genome (Fig. 3). The average contribution of D’ in each chromosome varied, while chromosome 2D was the least contributor and chromosome 7D the highest contributor (Fig. 3). For the majority of chromosome regions, the proportion of the D’ versus the D genome contribution was less than 18%. However, some chromosome regions were clearly more likely to have D’ alleles over D with a proportion equal to or greater than 50% (Fig. 3). The Supplementary Table S2 provides list of genes retrieved form RefSeqV1.1^66^ in regions with a higher likelihood to retain D’ genome alleles.

### Average genetic contribution to international nursery lines and released cultivars

**Figure 4:**
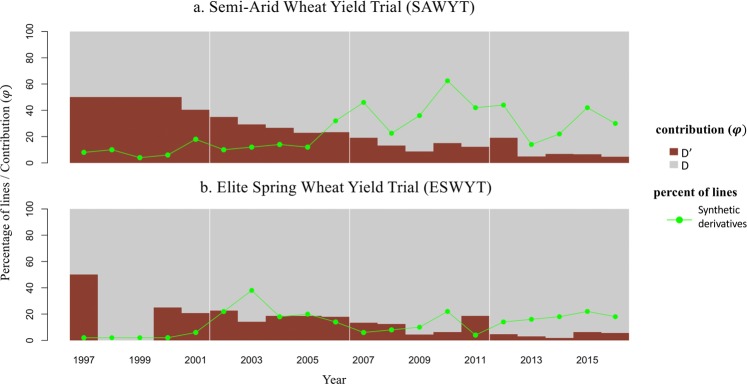
Theoretical contribution, *φ* and number synthetic derivative lines in two CIMMYT international yield trials SAWYT and ESWYT.

We also calculated the theoretical contribution of D’ in two international yield trials and in released cultivars with SH wheat in its pedigree. The average contribution of the D’ genome in synthetic derivative lines was generally higher in the Semi-Arid Wheat Yield Trial (SAWYT) than in the Elite Spring Wheat Yield Trial (ESWYT) (Fig. 4). In both trials, increasing number of synthetic derivatives with yearly fluctuations were retained in recent years. However, the contribution from the D’ genome decreased in recent years indicating that subsequent crosses have reduced the D’ genome contribution only retaining the favorable D’ regions in the genome. Across both international yield trials, 25 SH wheat was successfully utilized in developing competitive synthetic derivatives (Table 1). The SH lines ‘ALTAR 84/AEGILOPS SQUARROSA (TAUS)’ and ‘CROC_1/AEGILOPS SQUARROSA (224)’ were the most frequent SH wheat used. The synthetic derived lines, such as SOKOLL (PASTOR/3/ALTAR 84/AEGILOPS SQUARROSA (TAUS)//OPATA) and VOROBEY (CROC_1/AEGILOPS SQUARROSA (224)//OPATA/3/PASTOR) were subsequently used as parents.

**Table 1:** Synthetic hexaploid wheat and their contribution to synthetic derivative lines from international yield trials (ESWYT and SAWYT).

SN	Yield trial	Primary Synthetics	Number of SD	Average D’ Contribution *φ*	Nursery Number
1	SAWYT	ALTAR 84/AE.SQ	4	14.1	9,14,18,19
2	SAWYT	ALTAR 84/AE.SQUARROSA (191)	1	6.3	15
3	SAWYT	ALTAR 84/AE.SQUARROSA (205)	2	3.3	16
4	ESWYT	ALTAR 84/AE.SQUARROSA (211)	1	12.5	26
5	SAWYT	ALTAR 84/AE.SQUARROSA (219)	2	12.5	18
6	ESWYT	ALTAR 84/AE.SQUARROSA (221)	3	2.1	28,33,34
6	SAWYT	ALTAR 84/AE.SQUARROSA (221)	1	25.0	12
7	SAWYT	ALTAR 84/AE.SQUARROSA (224)	1	12.5	13
8	ESWYT	ALTAR 84/AEGILOPS SQUARROSA (TAUS)	12	9.2	31,36,37
8	SAWYT	ALTAR 84/AEGILOPS SQUARROSA (TAUS)	90	16.6	5–10,13–15,17–20,22–24
9	ESWYT	CENTURY/(TR.TA)TA-2450*	31	3.7	24,29–31,33–36
9	SAWYT	CENTURY/(TR.TA)TA-2450*	10	4.8	17,19,21–23
10	ESWYT	CHEN/AE.SQ	1	1.6	30
10	SAWYT	CHEN/AE.SQ	13	10.7	9,14–17,21,23,24
11	ESWYT	CHEN/AEGILOPS SQUARROSA (TAUS)	21	19.8	22–26,29–31
11	SAWYT	CHEN/AEGILOPS SQUARROSA (TAUS)	10	18.0	10,11,15,18,19,23
12	ESWYT	CNDO/R143//ENTE/MEXI_2/3/AEGILOPS SQUARROSA (TAUS)	13	9.8	23,24,26,30,31,33,35,36
12	SAWYT	CNDO/R143//ENTE/MEXI_2/3/AEGILOPS SQUARROSA (TAUS)	24	20.4	5,6,8,10–13,15–18,20–23
13	ESWYT	CROC_1/AE.SQUARROSA (205)	22	20.7	18,23–27,31,34,37
13	SAWYT	CROC_1/AE.SQUARROSA (205)	16	17.3	6,9,10,15–18,22,24
14	ESWYT	CROC_1/AE.SQUARROSA (213)	8	9.8	27,28,33,37
14	SAWYT	CROC_1/AE.SQUARROSA (213)	11	15.9	12,14,18,21,23,24
15	ESWYT	CROC_1/AE.SQUARROSA (224)	9	11.3	25,26,31,32,36,37
15	SAWYT	CROC_1/AE.SQUARROSA (224)	37	24.4	9,10,12–17,19–21,24
16	ESWYT	CROC-1/AE.TA(WX-224)	2	25.0	25,29
16	SAWYT	CROC-1/AE.TA(WX-224)	6	20.8	19,20,21,22
17	ESWYT	D67.2/PARANA 66.270//AE.SQUARROSA (320)	2	28.1	32,36
17	SAWYT	D67.2/PARANA 66.270//AE.SQUARROSA (320)	9	32.6	15,18,20,24
18	ESWYT	DVERD_2/AE.SQUARROSA (214)	1	25.0	21
19	ESWYT	DVERD_2/AE.SQUARROSA (221)	1	25.0	28
20	ESWYT	KS-8010-71/(TR.TA)TA-2470	4	6.3	24
21	SAWYT	SCA/AE.SQUARROSA (409)	1	25.0	15
22	SAWYT	T.DICOCCON PI225332/AE.SQUARROSA (895)	1	18.8	20
23	SAWYT	T.DICOCCON PI94625/AE.SQUARROSA (372)	1	12.5	19
23	SAWYT	T.DICOCCON PI94625/AE.SQUARROSA (372)	8	18.0	16,17,19,20
24	ESWYT	WICHITA/TA-1675(TR.TA)	2	4.7	24
25	SAWYT	YAR/AE.SQUARROSA (783)	1	6.3	24

*direct cross.

Among known released cultivars with synthetic background, the released cultivars retained an average of 17.48% of D’ genome contribution (Table 2). Noteworthy, cultivars with as high as 50% of D’ contribution (synthetic derivative lines of generation 1) have been released, although the majority of cultivars had less than 12.5% of D’ contribution (Supplementary Table S2).

**Table 2:** The average theoretical contribution of the D’ genome in international yield trials and synthetic derivative lines (SD) released as cultivars globally.

Germplasm^a^	Cycle	Number of samples	Number of SD	Average D’ contribution (*φ*)^b^
SAWYT	5–24	980	249	18.43
ESWYT	18–37	1,000	133	12.12
Released Cultivars	—	—	62	17.48

^a^ESWYT (Elite Spring Wheat Yield Trial), SAWYT (Semi-arid Wheat Yield Trial).

^b^average theoretical contribution in percent.

## Discussion

Our study provides a unique perspective of the genetic contribution of *Ae*. *tauschii* to the CIMMYT spring bread wheat breeding program via SH wheat, which have been of interest for many years^23^. Studies have shown that the D genome diversity of SH wheat is considerably higher than of bread wheat^25,27^. Although direct hybridization of bread wheat with *T*. *turgidum* and *Ae*. *tauschii* is possible, it is generally difficult to generate and maintain stable genomes^67^. As a result, the development of SH wheat is considered a better alternative. In addition, SH wheat can also serve as bridge to introduce alleles from tetraploid durum wheat and emmer wheat to hexaploid bread wheat^33^. In contrast to initial years when few elite durum wheat genotypes were used to develop SH, in more recent years additional lines from the tetraploid *Triticum dicoccoides* and *Triticum dicoccum*^20,23,68^ pools were introgressed.

The potential of wild relatives of wheat including *Ae*. *tauschii* to improve disease resistance in bread wheat is well documented. For example, the leaf rust resistance gene *Lr2*1 was introgressed into the wheat cultivar Thatcher from *Ae*. *tauschii* accession TA1599 via SH wheat^69^. Similarly, gene *Yr28* and other genes of resistance to stripe rust have been transferred from *Ae*. *tauschii*^70^. More recently, two *Ug99* stem rust resistance genes (*SrTA10187* and *SrTA10171*) were transferred from *Ae*. *tauschii* into the hard-white winter wheat line KS05HW14^71^. In several instances, synthetic derivative lines have also been reported to positively contribute to yield and abiotic stress torelance^19,72–74^.

Beside *Ae*. *tauschii*, other wild wheat relative species can be introgressed into wheat without development of synthetics. However, usually the transfer of the alien segments is practically and methodologically more challenging and creates a situation where no homologous pairing occurs. The D’ genome being the progenitor of the D genome, problems of homologous paring have not been reported. Due to its allo-hexaploid nature, bread wheat is in general have high buffering capacity to alien transfers.

The A and B genomes are more closely related with each other than the A *vs*. D genome or the B *vs*. D genome^5,6^. This makes it more difficult to find SNP markers specific to the A or B genome. The A and B genomes from durum and bread wheat have common ancestors, while natural hybridization between the genomes occurred 8,000 years ago. Both genomes have gone through natural and artificial selection over years, thus likely evolved in a similar manner. Moreover, the dwarfing gene *Rht1* in modern durum wheat was transferred to bread wheat further allowing the mixing of the two species. Our study clearly showed that the differences between the A or B genomes from durum and bread wheat were smaller than between the D’ and D genomes.

The theoretical contribution of the D’ genome was a good predictor of the estimated marker-estimated D’ genome contribution. However, synthetic derivatives within each theoretical contribution class (breeding cycles 1 to 7) differed significantly for the estimated marker- contribution and for genomic regions deriving from the D’ genome. Thus, the theoretical estimates provide some indication about the potential contribution of D’, but is not an accurate reflection due to natural and artificial selection, linkage drag and Mendelian sampling that occurs during the breeding process.

Adaptive introgression is very common phenomena, which leads to unbalanced contributions in different part of the genome particularly in regions vital for plant survival and adoption^75^. SH wheat shows a number of undesirable traits such as a shattering rachis, glume retention, glume hardiness and tall plant height^33,67^. Therefore, during the process of developing synthetic derivatives, large progeny populations (exceeding 2,000 to 3,000 plants in size) were grown to select for agronomically desirable traits particularly threshability, plant height and disease resistance^33^. Natural adaption and adaptive introgression apply as some progenies do not survive due to chlorosis or necrosis. In addition, the fitness advantage of wild or domesticate alleles may be favored in process of natural selection^76–78^.

The large progeny populations are used by breeders for stringent selection. The probability of retaining D’ is higher in regions surrounding QTL/genes of interest when certain plant types and target traits are selected and can last several generations of backcrossing^21^. Our results showed a larger extent of the residual D’ contribution was possibly due to linkage drag in later breeding cycles. We also demonstrated that there is no clear biased preference among alleles in the majority of genome regions. However, some bias in contribution is obvious as during the process of developing the synthetic derivatives both natural and artificial selection are applied, possibly favoring genes related to survival and to typical bread wheat plant types. The search for the underlying genes in the preferable selected D’ genome segments did not provide any deeper insight as the function of most genes was unknown.

CIMMYT established its Wide Crosses Program, in 1986. Since their development, synthetic derivative lines were then frequently used in mainstream breeding and the superior progenies were selected for inclusion and distribution through international yield trials and nurseries, a few were released as cultivars. In the past 32 years, CIMMYT has generated more than 1,401 spring type SH wheat and over two thousand crosses made between the most promising SH wheat and elite bread wheat lines. Generating winter type SH wheat started in 2008. Approximately 50 targeted SH wheat are currently developed by CIMMYT annually, by crossing current elite durum wheat cultivars, but extending the genetic diversity of the *Ae*. *tauschii* accessions by using combined genotypic and phenotypic information.

The number of SH wheat increased in both yield trials (ESWYT and SAWYT) during the last 19 years, however there was a declining trend of the D’ genome contribution on an individual basis. Synthetic derivatives of breeding cycle 1 with a D’ genome contribution of 50% formed part in yield trials disseminated from 1997 to 2000, whereas in more recent years the average D’ genome contribution was less than 15%. Although 1524 SH wheat were developed to date, derivatives from 25 SH wheat reached the yield trials. Over one thousand initially developed SH wheat were characterized for agronomic and other economically important traits and below 10% were found to have potential for use in breeding programs; the set is available as Elite Synthetics from the CIMMYT germplasm bank. Similarly, thousands of synthetic derivatives have been characterized over the years, some now released as varieties, however the most popular synthetic derivatives, e.g. SOKOLL and VOROBEY, were used multiple times as parents in mainstream breeding due to their superior performance under drought stress and resistance to *Septoria tritici* blight. The SH wheat ALTAR_84/AEGILOPS SQUARROSA(TAUS) was the most frequent line in both ESWYT and SAWYT. Among the durum wheat donors ALTAR_84 dominated followed by CROC_1 (=LAHN) and CHEN. ALTAR_84 was bred during the 1980s based on a new ideotype concept, with balanced increase in all yield components and was extensively grown in Mexico. The higher percentage of SH wheat in the SAWYT is due to their potential introgression of new stress tolerance genes^79^. A more recent outcome of the utilization of SH has been the development and release of high grain Zn, biofortified, varieties ‘Zn Shakti’ from a durum wheat SH, and ‘WB-02’, ‘PBWZn-1′, and ‘Nohely F2018’ from *T*. *dicoccum* based SH wheat.

The value of introgression of SH wheat is also demonstrated by an increasing number of varieties released in various countries. It is difficult to estimate the use of synthetic derivative lines as parents across breeding programs due to the (a) unavailability of pedigree record of cultivars released in different countries and (b) incomplete or inaccurate pedigree records available. Based on available pedigree records some cultivars are released with as high as 50% theoretical D’ genome contribution (e.g. KT2009 in Pakistan). This means the buffering capacity of the A and B genome can mask undesirable effects of wild alleles.

The development of SH wheat led to increased D genome diversity via the introgression of D’ at CIMMYT. However, for SH wheat to be increasingly used in mainstream breeding, a targeted introgression of high value traits combined with a proper selection strategy through limited crossing is considered important to capture additional useful genes/diversity from SHs. The breeding strategy needs to consider minimizing the unfavorable contribution of the D’ genome due to linkage drag while maintaining the desirable alleles. This raises the question, how much of D’ genome contribution is acceptable or favorable in synthetic derivatives or what is the average percentage of D’ genome contribution that must be discarded using selection. The theoretical contribution in the most recent CIMMYT international yield trials remained less than 12.5% contribution of the D’ genome which means that at least three rounds of crosses with elite bread wheat lines is required if backcrossing and stringent selection for multiple traits is not applied. As we have seen the difference between the theoretical and marker-estimated contribution of D’ at the individual and across genome level, accelerating the process through an appropriate breeding approach can only be archived by getting rid of the unnecessary genome segments of D’ while retaining desirable genes/QTLs. Combining genomic profiles with the phenotypic assessment of SH wheat could help to identify the desirable genes/QTL, which could be traced during the selection process via genomic-assisted breeding approaches.

## Conclusion

The results show that the genetic contribution of the D’ genome of *Ae*. *tauschii* can be estimated using high-density genome profiles. The marker-estimated contribution of D’ in this study underlines the importance of SH wheat in maintaining diversity and genetic gain over years. However, targeted breeding efforts are required to effectively use the D’ diversity for mining desirable alleles. Molecular markers, if tagged to desirable alleles, will have the potential to accelerate the selection process to maintain the desirable variation while culling the undesirable variation. Overall, the development and utilization of SH wheat at large scale at CIMMYT is a model for maintaining diversity in bread wheat breeding germplasm required for combating future challenges in crop improvement and is a successful example of the utilization of genetic diversity from wild relatives.

## Methods

**Figure 5:**
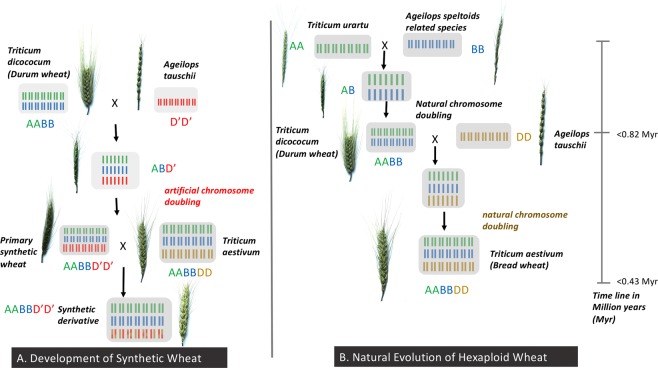
Development of synthetic hexaploid wheat (AABBDD’) in comparison to the emulating evolution of the hexaploid wheat (AABBDD).

The SH wheat included in this study were developed by CIMMYT wide-crosses program by crossing durum or emmer wheat (*Triticum dicoccum*, AABB) with *Ae*. *tauschii* (D’D’), followed by artificial doubling of the chromosomes of resulting haploid F_1_ plants (Fig. 5, Supplementary Fig. S5). The SH wheat was then crossed with bread wheat lines via 1 or 2 backcrosses or 3-way (top) crosses, to generate advanced lines through selections conducted in segregating generations and as fixed lines; known as a synthetic derivative line (Supplementary Fig. S5). The SH wheat crossed “x” times with bread wheat is termed as synthetic derivative line of breeding cycle “x”. For example, synthetic derivative line of breeding cycle 1 is defined by an SH wheat crossed once with a bread wheat.

### Marker-based contribution estimation

A total of 359 genotypes used in this study included three *Ae*. *tauschii* lines, 30 durum wheat lines, eight SH wheat, 253 synthetic derivative lines, and 63 bread wheat lines (Supplementary Table S3). Synthetic derivative lines were between 1 to 7 cycles in derivation. The synthetic derivative lines were selected because of their relevance in the CIMMYT Spring Bread Wheat Improvement Program, with at least one sister line distributed through CIMMYT international yield trials, but with at least 3 to 4 sister lines conserved in the CIMMYT Wheat Germplasm Bank.

All entries were genotyped with the DArTseq® technology at the Genetic Analysis Service for Agriculture (SAGA) laboratory at CIMMYT in Mexico. A complexity reduction method including two enzymes (*PstI* and *HpaII*) was used to create a genome representation of the set of samples. A *PstI*-RE site specific adapter was tagged with 96 different barcodes enabling multiplexing a 96-well microtiter plate with equimolar amounts of amplification products within a single lane on an Illumina HiSeq2500 instrument (Illumina Inc., San Diego, CA). The successful amplified fragments were sequenced up to 77 bases, generating approximately 500,000 unique reads per sample. A proprietary analytical pipeline developed by DArT P/L was used to generate allele calls for SNP and SilicoDArT. Then, a set of filtering parameter was applied to select high quality markers for this specific study. A total 76,543 Single Nucleotide Polymorphism (SNPs) and 365,663 SilicoDArT were revealed. SilicoDArT markers are a secondary marker type provided by DArTseq®, which are scored for the presence or absence of a single loci. The genotypic data were then filtered based on the following consecutive steps:

- Markers with known chromosome position on the Chinese Spring reference genome sequence RefSeq v1.0^66^ (number of markers retained = 211,169). Markers with unknown genomic position were removed.
- Markers genome specific for the A, B or D genomes, respectively (number of markers retained = 202,668).
- Markers with a missing data rate less than 30% (number of markers retained = 188,584)
- Markers with minor allele frequency MAF ≥ 0.05 and heterozygote frequency HET ≤ 0.1 (number of markers retained = 37,201)
- Markers with a population specific allele differential greater than 0.30 (see equation 1 and 2, below) (number of markers retained for D genome = 2,669). Such arbitrary threshold was set to reduce number of markers that are most informative. To determine a marker-estimated genetic contribution of the D’ genome in the selected set of synthetic derivative lines, we only used markers with a large allele-frequency difference among the populations, i.e. markers with population-specific alleles^80,81^.

The primary populations in our study included the *Ae*. *tauschii* - SH wheat (*shw*), bread wheat (*bw*), durum wheat (*dw*) lines, where geneflow across the populations rarely occurs, whereas the secondary population consisted of the synthetic derivative lines (*sd*) which were derived by the introgression of *shw*, *bw* and *dw*, respectively. We assumed a SNP marker with two alleles (1 and 2) at locus L with an allele frequency *P*_*1*shw_, *P*_*2shw*_ in the *Ae*. *tauschii –* SH wheat population, and *P*_*1bw*_, *P*_*2bw*_ in the bread wheat population and *P*_*1sd*_, *P*_*2sd*_ in the synthetic derivative line population. Then the population specific differential (δ_D_) can be represented as:

1 $${\delta }_{{\rm{D}}(l)}=|{P}_{1shw}^{(l)}-{P}_{2bw}^{(l)}|=|{P}_{2shw}^{(l)}-{P}_{2bw}^{(l)}|$$

For calculating population specificity at A and B, let allele frequency P_1dw_, P_2dw_ for durum wheat population, the population specific differential (δ_A or B_) can be represented as:

2 $${\delta }_{{\rm{A}}or{\rm{B}}(l)}=|{P}_{1dw}^{(l)}-{P}_{2bw}^{(l)}|=|{P}_{2dw}^{(l)}-{P}_{2bw}^{(l)}|$$

For calculation of D’ contribution, the minimum population specific differential (*δ*_*D*(*l*)_) was set to greater than 0.30 as 80% of the markers had the differential less than 0.30^82^. Using the allele frequencies, the least-square estimation of the introgression proportion (*ϕ*), as a measure of the genetic contribution for D’ at each locus *l*^83,84^ can be expressed as

3 $${\varphi }_{l}=\frac{{\sum }_{i=1}^{2}({P}_{shw}^{(l)}-{P}_{bw}^{(l)})({P}_{sd}^{(l)}-{P}_{bw}^{(l)})/{P}_{sd}^{(l)}}{{\sum }_{i=1}^{2}{({P}_{shw}^{(l)}-{P}_{bw}^{(l)})}^{2}/{P}_{sd}^{(l)}}$$

and overall L loci:

4 $$\varphi =\frac{{\sum }_{l=1}^{L}{\sum }_{i=1}^{2}({P}_{shw}^{(l)}-{P}_{bw}^{(l)})({P}_{sd}^{(l)}-{P}_{bw}^{(l)})/{P}_{sd}^{(l)}}{{\sum }_{l=1}^{L}{\sum }_{i=1}^{2}{({P}_{shw}^{(l)}-{P}_{bw}^{(l)})}^{2}/{P}_{sd}^{(l)}}$$

Then sampling variance of $\varphi$ is: ${\rm{V}}(\varphi )=\frac{MSE}{{\sum }_{l=1}^{L}{\sum }_{i=1}^{2}{({P}_{shw}^{(l)}-{P}_{bw}^{(l)})}^{2}\,/{P}_{sd}^{(l)}}$, where $MSE=\frac{{\sum }_{l=1}^{L}{\sum }_{i=1}^{2}{[({P}_{sd}^{(l)}-{P}_{bw}^{(l)})(\varphi {P}_{shw}^{(l)}-{P}_{bw}^{(l)})]}^{2}\,/{P}_{sd}^{(l)}}{r-L}$ ${\rm{and}}\,r={{\sum }_{l=1}^{L}r}_{l}$ is the total number of alleles at all L loci.

The D’ contribution at each locus was plotted across the wheat chromosome to determine if the contribution is disproportionate in different segments of the genome. The proportion of contribution from the parental populations, D and D’, were calculated using the admixture 1.3 program that employs the maximum likelihood approach^85,86^ under supervised learning mode where reference individuals are members of the SH and bread wheat populations and leaving the synthetic derivative lines as unknown degree of contribution from the two parental populations.

### Theoretical contribution estimation

The theoretical contribution of D’ to synthetic derivative lines was calculated using the available pedigree information, where pedigrees were extended at multiple levels and were traced back to the first cross between the SH wheat and bread wheat using the International Wheat Information System (IWIS) version 2 (https://www.cimmyt.org/funder_partner/international-wheat-improvement-network-iwin/). IWIS, curated by CIMMYT, has a collection of pedigree and phenotypic data recorded since 1976 to date. Using a recursive approach using the pedigree information, we calculated the theoretical contribution of D’. The *φ*_*I*_, the D’ contribution to individual i can be estimated with D contribution to first parent (female), abbreviated as *φ*_*P*1_, and second parent (male), *φ*_*P*2_, with the formula:

5 $${\phi }_{I}=\frac{1}{2}({\phi }_{P1}+{\phi }_{P2})\times 100$$

As SH wheat has the entire D’ genome derived from *Ae*. *tauschii*, the *φ*_*I*_ is considered as 100. For bread wheat with no SH wheat in its pedigree, the *φ*_*I*_ is considered as 0. The different accessions of *Ae*. *tauschii* were assumed to have equal amounts of contribution. The theoretical and marker-estimated contribution of D’ was compared across all included synthetic derivative lines.

### Average genetic contribution to international nurseries and released cultivars

To estimate the impact of SH wheat in the CIMMYT spring bread wheat breeding program the average theoretical contribution of D’ was estimated in two international yield trials, which were annually disseminated to 100–400 national partners worldwide as part of the CIMMYT International Wheat Improvement Network (IWIN). The entries of the Elite Spring Wheat Yield Trial (ESWYT) targeted to irrigated wheat growing areas and the Semi-Arid Wheat Yield Trial (SAWYT) targeted to rain-fed areas from 19 years (1997 to 2016) were used (Table 2). The entries included in these two yield trials are considered to be the best CIMMYT breeding lines for their target environment testing and up-take as cultivars in the developing world. The average contribution of D’ in synthetic derivative lines released as cultivars worldwide between 2003–2017 were also analyzed (Table 2, Supplementary Table S2). All calculations and plotting, otherwise specified above, were performed in R^87^.

## Supplementary information

Supplementary Figures S1 to S5

Supplementary Tables S1 to S5

## Data Availability

The data is available at CIMMYT Dataverse (http://hdl.handle.net/11529/10548269).
