# Supplementary material for: Genetic Contribution of Synthetic Hexaploid Wheat to CIMMYT’s Spring Bread Wheat Breeding Germplasm
Source: Sci Rep. 2019 Aug 26;9:12355. doi: 10.1038/s41598-019-47936-5 (PMC6710277; doi:10.1038/s41598-019-47936-5)
Supplement: Supplementary file 1 — Supplementary Figures S1 to S5 [file 41598_2019_47936_MOESM1_ESM.pdf]

Supplementary Figures

Genetic Contribution of Synthetic Hexaploid Wheat to CIMMYT's Spring Bread Wheat Breeding Germplasm

Umesh Rosyara, Masahiro Kishii, Thomas Payne, Carolina Paola Sansaloni, Ravi Prakash Singh, Hans-Joachim Braun, Susanne Dreisigacker

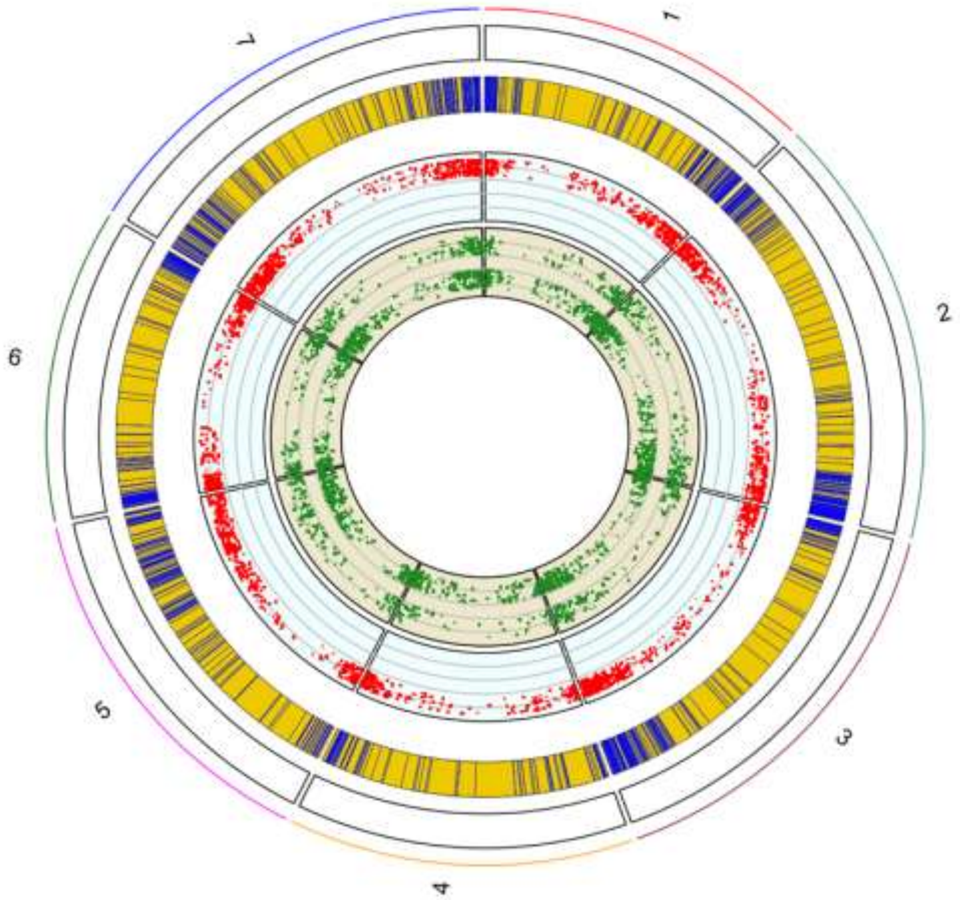

Figure S1: SNP markers (2669) and their distribution in the D genome – from outside: track 2 positions of PAV and SNPs, track 3: call rate of each marker (red dots), track 4: frequency difference between the *Ae. tauschii*/synthetic hexaploid wheat population and bread wheat population (green dots).

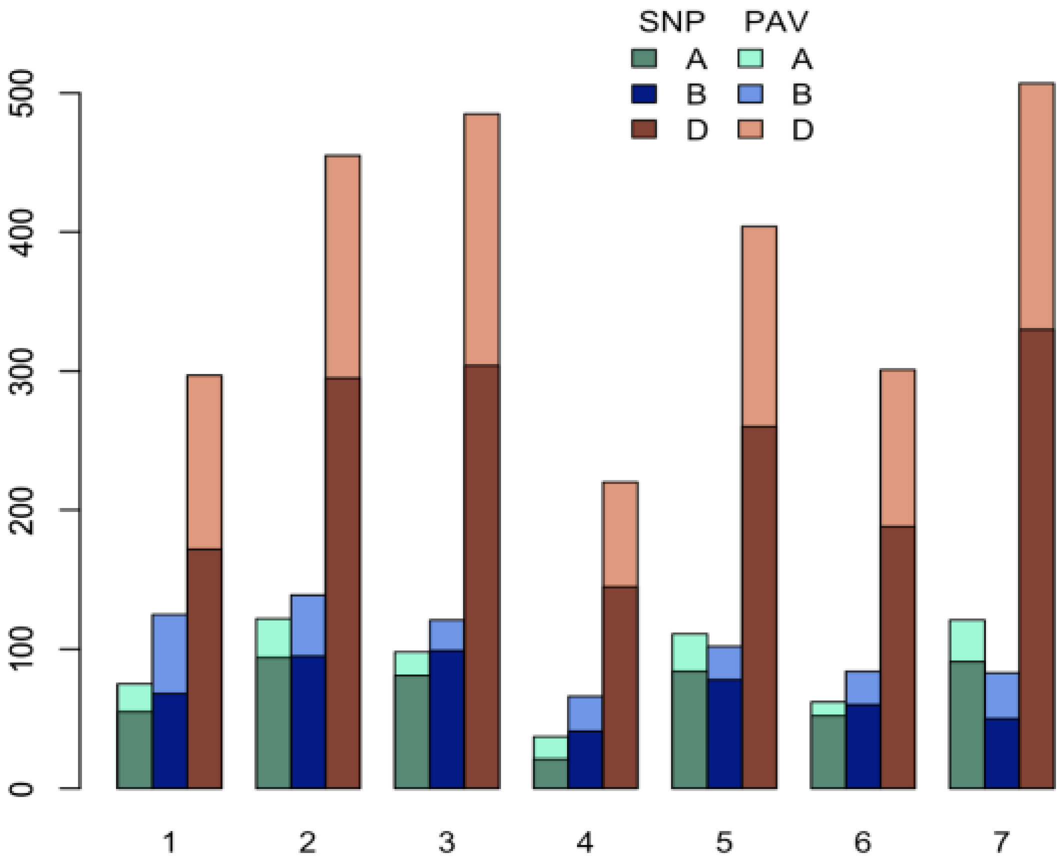

Figure S2: Number of genome specific markers with allele frequency difference greater than 0.3 among populations. Number of markers in genomes A and B are based on allele frequency different between durum wheat (AABB) and bread wheat (AABBDD) populations whereas the number of markers in the D genome are based on allele frequency difference between bread wheat (D) and *Ae. tauschii* - synthetic hexaploid wheat (D') populations.

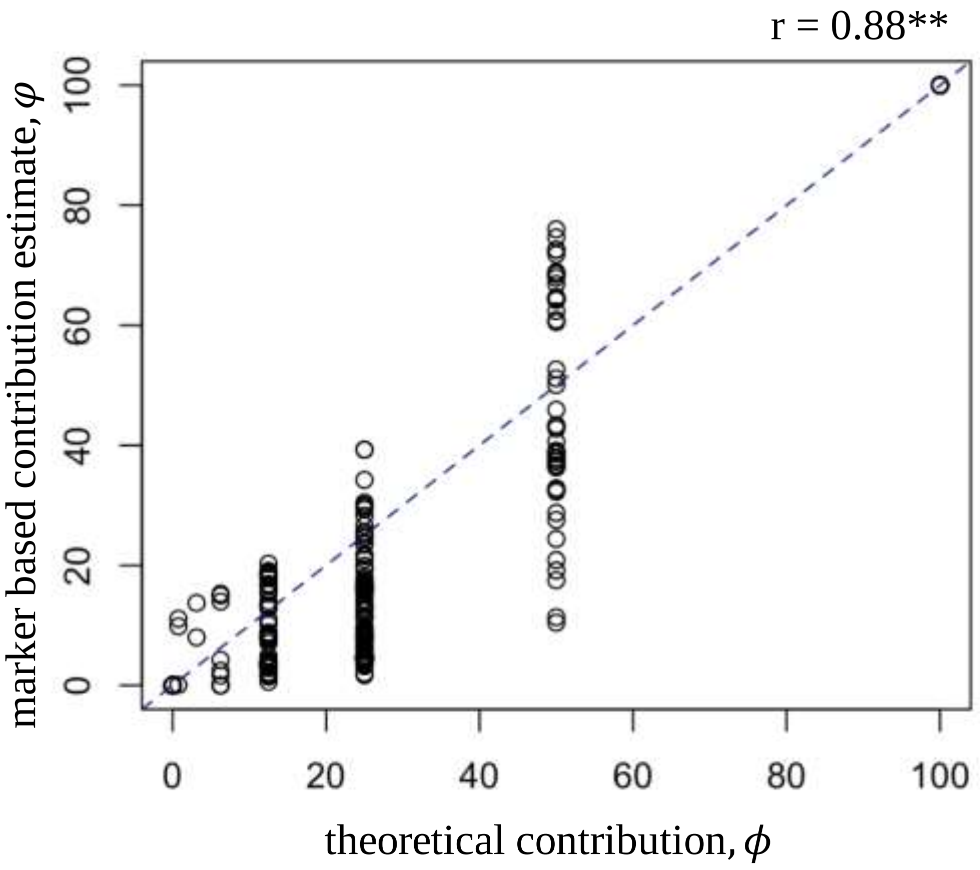

Figure S3: Comparison of theoretical contribution estimates versus marker-based contribution estimates

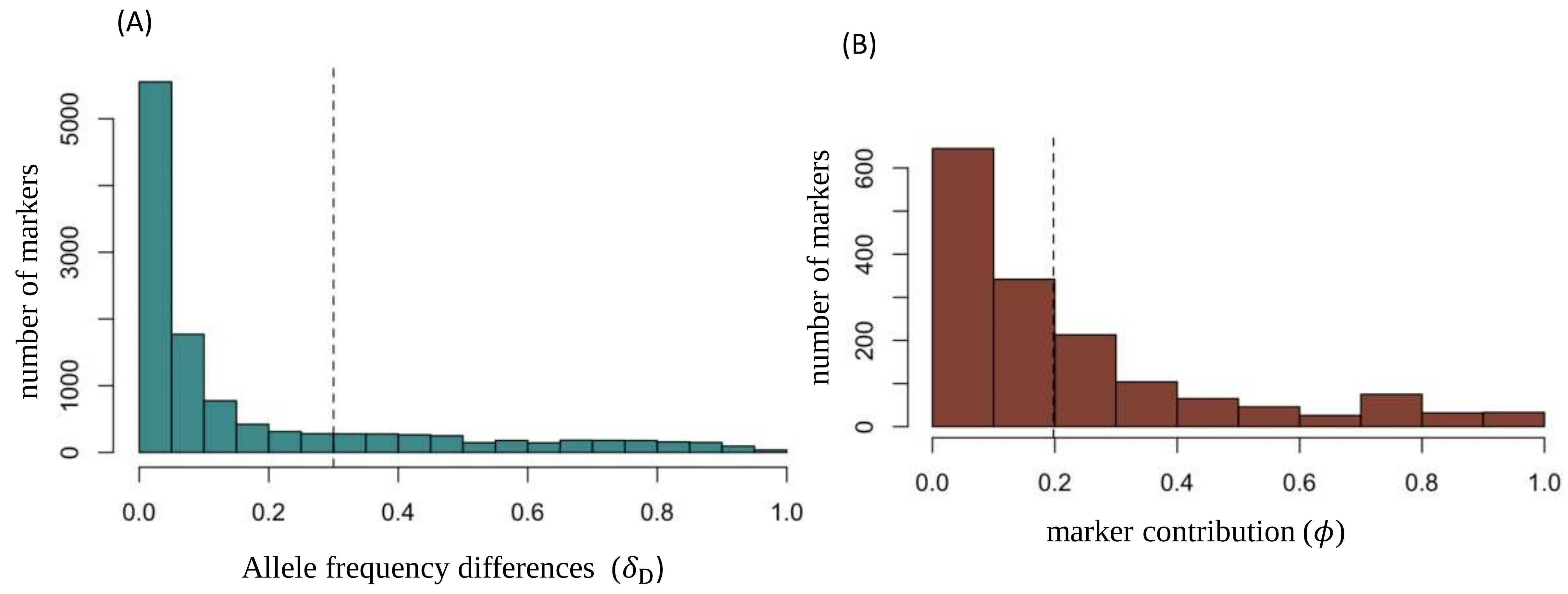

Figure S4: (A) Allele frequency differences (δ_D) distribution of all useful D markers and (B) marker contribution (ϕ) for selected markers with $\delta_D > 0.30$

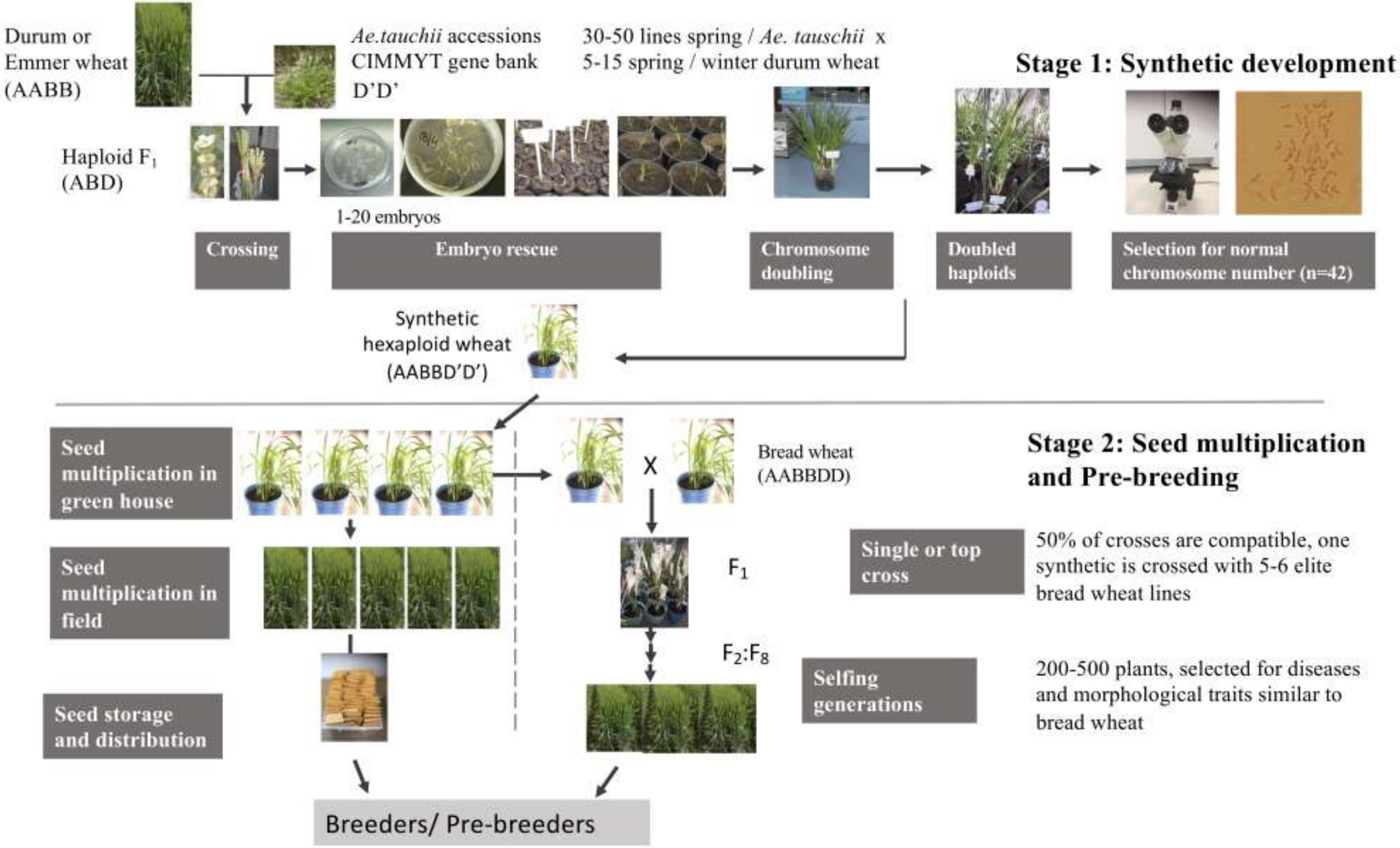

Figure S5: Synthetic hexaploid wheat and their derivative development protocol followed by CIMMYT wide-crosses program
